# Exploring healthcare professionals’ views of the acceptability of delivering interventions to promote healthy infant feeding practices within primary care: a qualitative interview study

**DOI:** 10.1017/S1368980020004954

**Published:** 2020-12-15

**Authors:** Elaine Toomey, Caragh Flannery, Karen Matvienko-Sikar, Ellinor K Olander, Catherine Hayes, Tony Heffernan, Marita Hennessy, Sheena McHugh, Michelle Queally, Patricia M Kearney, Molly Byrne, Caroline Heary

**Affiliations:** 1 Health Behaviour Change Research Group, National University of Ireland Galway, Galway, Ireland; 2 School of Public Health, University College Cork, Cork, Ireland; 3 School of Health Sciences, City, University of London, London, UK; 4 School of Medicine, Trinity College Dublin, Dublin, Ireland; 5 Mallow Primary Healthcare Centre, Co. Cork, Ireland; 6 School of Economics, National University of Ireland Galway, Galway, Ireland; 7 School of Psychology, National University of Ireland Galway, Galway, Ireland; 8 School of Allied Health, University of Limerick, Limerick, Ireland

**Keywords:** Infant feeding, Early-life nutrition, Childhood obesity, Primary care, Prevention, Intervention

## Abstract

**Objective:**

Early-life nutrition plays a key role in establishing healthy lifestyles and preventing chronic disease. This study aimed to (1) explore healthcare professionals’ (HCP) opinions on the acceptability of and factors influencing the delivery of interventions to promote healthy infant feeding behaviours within primary care and (2) identify proposed barriers/enablers to delivering such interventions during vaccination visits, to inform the development of a childhood obesity prevention intervention.

**Design:**

A qualitative study design was employed using semi-structured telephone interviews. Data were analysed using qualitative content analysis; findings were also mapped to the Theoretical Framework of Acceptability (TFA).

**Setting:**

Primary care in Ireland

**Participants:**

Twenty-one primary care-based HCP: five practice nurses, seven general practitioners, three public health nurses, three community dietitians and three community medical officers.

**Results:**

The acceptability of delivering interventions to promote healthy infant feeding within primary care is influenced by the availability of resources, HCP’s roles and priorities, and factors relating to communication and relationships between HCP and parents. Proposed barriers and enablers to delivering interventions within vaccination visits include time constraints *v*. opportunistic access, existing relationships and trust between parents and practice nurses, and potential communication issues. Barriers/enablers mapped to TFA constructs of Affective Attitude, Perceived Effectiveness and Self-Efficacy.

**Conclusions:**

This study provides a valuable insight into HCP perspectives of delivering prevention-focused infant feeding interventions within primary care settings. While promising, factors such as coordination and clarity of HCP roles and resource allocation need to be addressed to ensure acceptability of interventions to HCP involved in delivery.

The period from pregnancy through to the first 2 years of life represents an important stage in a child’s development and is characterised by the rapid growth and development of organs, systems and behaviours^(1,2)^. The importance of early intervention to establish healthy eating behaviours and optimum infant nutrition is increasingly recognised for the promotion of overall health and the prevention of chronic conditions such as childhood obesity^(3)^. Childhood obesity is associated with increased risk of CVD^(4)^, diabetes^(4)^ and certain forms of cancer as well as long-term morbidity and pre-mature mortality and is a significant global health concern^(5)^.

Potentially modifiable infant feeding behaviours linked with later development of childhood obesity include the initiation and duration of breast-feeding, and the timing and type of solid food introduction (i.e. complementary feeding)^(3,6–12)^. The WHO recommends infants should be breastfed exclusively for the first 6 months of life, with nutritionally adequate and appropriate solid foods introduced subsequently^(13)^. However, international evidence suggests that this is not happening. According to the UNICEF global database, almost one-third of infants aged 4–5 months have been introduced to solid foods, with concerns also raised about the types of food provided^(14)^. A nationally representative US study showed that 16·3 % of infants had been introduced to complementary foods before 4 months^(15)^, and a recent study identified that 18 % of Irish infants began complementary feeding before 17 weeks^(16)^. Previous research has also shown that a substantial proportion of Irish infants aged 6 months consume foods high in saturated fats, salt and refined sugars^(17)^. As such, there is a clear need for the development and evaluation of interventions to improve infant feeding behaviours as a potential mechanism to prevent childhood obesity as well as contributing to positive health outcomes.

There is growing acknowledgement of the role of community-based public health interventions for prevention of childhood obesity, and of the potential benefits for involving community-based healthcare professionals (HCP)^(18–20)^. HCP are a trusted source of information and support for parents of infants^(21,22)^. In addition, primary care HCP have frequent contact with parents in the early years of life, often at times when the child is well, such as during routine check-ups or vaccination visits. However, there are several potential challenges to the delivery of childhood obesity prevention interventions by HCP within primary care settings. Although opportunistic interventions may offer promise in terms of the potential to ‘make every contact count’^(23)^, previous research has found that a lack of time and resources hinder the capacity for HCP to deliver opportunistic behaviour change public health interventions within paediatric and other routine care settings^(24–27)^. Specific HCP-level issues such as insufficient knowledge and skills in relation to childhood obesity have also been identified^(27–29)^. However, this research has often focused on issues relating to the *management* of childhood obesity^(26–28)^, rather than prevention, and explorations of opportunistic infant feeding interventions specifically are also limited^(29)^. Therefore, although infant feeding interventions to prevent childhood obesity delivered by HCP within routine primary care settings show promise, there is a need to specifically explore HCP’s opinions on the acceptability of such interventions^(30,31)^. Intervention acceptability is a ‘multi-faceted construct reflecting the extent to which people delivering or receiving a healthcare intervention consider it to be appropriate, based on anticipated or experienced cognitive and emotional responses’ and can be assessed from two temporal perspectives (i.e. prospectively or retrospectively)^(32)^. Prospective assessment of intervention acceptability is crucial to highlight factors which may influence the acceptability and overall success of an intervention and should be addressed during intervention development^(32)^.

This study aimed to prospectively explore primary care HCP’s opinions on the acceptability of delivering interventions to promote healthy infant feeding behaviours within primary care settings and proposed factors influencing this, drawing on their experience as primary care practitioners. The study also aimed to inform the development of the Choosing Healthy Eating for Infant Health (CHErIsH) intervention and to identify anticipated barriers and enablers to delivering infant feeding interventions during vaccination visits. The CHErIsH multidisciplinary study aims to develop and evaluate a brief intervention to support and promote healthy infant feeding practices delivered during vaccination visits, with an overall goal of preventing childhood obesity^(33,34)^.

## Methods

### Study design

A qualitative study design was employed using semi-structured interviews. The study was guided by critical realism^(35,36)^ to allow the experiences, meanings and realities of participants to be captured and interpreted. Written informed consent was obtained from each participant.

### Participants

Primary care HCP directly involved in healthcare provision for infants and who have routine contact with parents of infants were invited to take part in the study. Experience of delivering infant feeding interventions was not required. Specifically, purposive sampling was used to ensure representation from males and females, and from practice nurses, general practitioners (GP), community dietitians, public health nurses (PHN) and community/area medical officers. Participants were recruited via Twitter, word of mouth, snowball sampling and through invitation emails sent by professional organisations (Irish Practice Nurses Association, Irish Nutrition and Dietetics Institute and the Primary Care Trials Network Ireland).

### Data collection and reflexivity

A topic guide containing open-ended questions regarding HCP’s views on the acceptability of delivering infant feeding interventions within routine practice, their roles and potential barriers and enablers was developed by ET and KMS and piloted with a GP for content, terminology and appropriateness prior to data collection (Appendix 1). Semi-structured telephone interviews were conducted by ET, a full-time female researcher with experience of qualitative data collection, a clinical background in primary care service delivery and a key role in developing the CHErIsH intervention. This was made known to all study participants by way of introduction. Interviews were conducted over the phone for participant convenience and to allow participants a degree of anonymity to express their opinions. Only one participant was known to the lead author prior to the interviews. The importance of expressing honest opinions was made clear to participants from the outset. Interviews were audio-recorded and transcribed verbatim.

### Data analysis

Qualitative directed content analysis was conducted as informed by Hsieh and Shannon^(37)^ and also drawing on the work of Assarroudi et al^(38)^ Content analysis was chosen because it provides a flexible approach to identifying, analysing and reporting patterns across qualitative data and lends itself to a critical realist approach^(35)^. A combination of deductive and inductive approaches was used to facilitate practical and timely application of findings for informing CHErIsH intervention development. Interview transcripts were read and reread by ET for familiarisation. Key findings for each participant were initially summarised deductively according to the topic guide questions, and these summaries were collated to form initial codes. Line-by-line coding of raw data from all transcripts was then conducted which drew on these initial codes as well as inductively developing new codes. To strengthen rigour, credibility and facilitate reflexivity within the analysis, a second author (CF) coded two transcripts also drawing on initial codes, and subsequently discussed coding with ET^(37)^. Once all data had been coded, ET reviewed and sorted initial codes into categories by merging conceptually similar codes, and organising and grouping codes into meaningful clusters^(37,38)^. A descriptive narrative for each emergent category was then written by ET and reviewed by CF. Both authors met to discuss and refine the final groupings to ensure distinct and coherent categories and to identify and organise subcategories and exemplar quotes. Final categories represented the overarching key factors that influenced the prospective acceptability of delivering of infant feeding interventions within primary care according to HCP. Subcategories reflected either negative or positive aspects of these factors, for example, specific barriers and enablers. NVIVO 12 was used to manage data and facilitate the process, and an audit trail was kept to document the analysis process (Appendix 2).

**Fig. 1:**
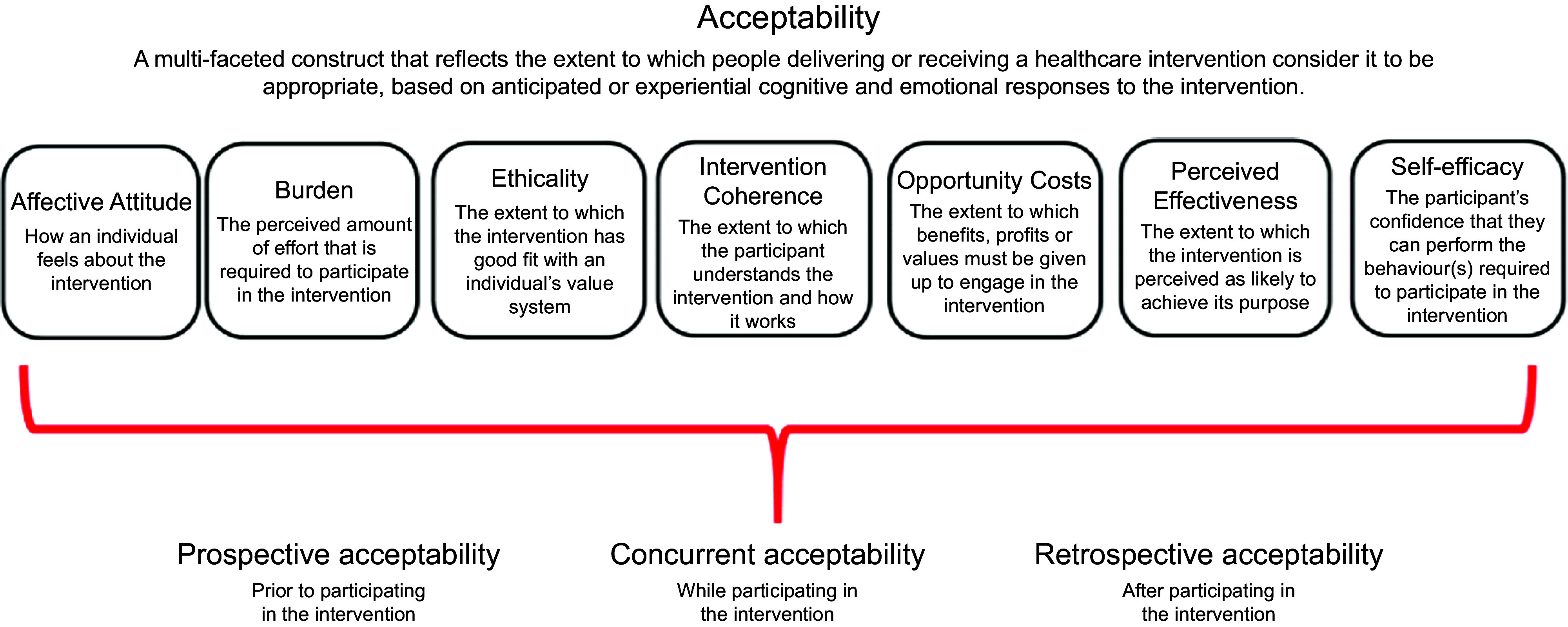
Constructs of the theoretical framework of acceptability. Reproduced with permission from Sekhon *et al.* (2017)^(37)^

As a key focus was to explore intervention acceptability, two authors (ET, EO) mapped the identified barriers and enablers to the Theoretical Framework of Acceptability (TFA)^(32)^. The TFA is a comprehensive method for assessing prospective and retrospective acceptability. The framework identifies seven conceptually distinct constructs (Fig. 1) that are proposed to capture key dimensions of acceptability. It was used to enhance the applicability of study findings by structuring findings according to recognised constructs of acceptability and to ensure that all constructs would be explicitly considered. We developed a codebook which defined TFA constructs within the context of this study (Appendix 3) and independently coded each barrier and enabler according to these constructs. Coding was then compared with discrepancies or ambiguities resolved through consensus discussion.

## Results

This study has been reported in accordance with the Consolidated criteria for reporting qualitative research (COREQ) criteria (Appendix 4).

### Participants

Twenty-one HCP from a variety of primary care disciplines were interviewed (Table 1). Interviews lasted on average of 33 min, ranging from 23 to 53 min.

**Table 1:** Participant characteristics

Variables	*n*	%
Gender		
Female	18	86
Male	3	14
Profession		
General practitioner	7	33
Practice nurse	5	24
Public health nurse	3	14
Community dietitian	3	14
Community medical officer	3	14
Variables		
Age		
Mean/median	44/42	
Range	29–60	
Length of time qualified (years)		
Mean/median	21/19	
Range	2–40	
Length of time working in primary care (years)		
Mean/median	12/11	
Range	2–36	
Has children (Y:N)	20:1	

### Summary of findings

In general, HCP felt that primary care provides good access to parents of infants and opportunities to address infant feeding. However, based on their experience, HCP identified several factors influencing the prospective acceptability of delivering interventions to promote healthy infant feeding in primary care settings (Table 2). Factors were broadly categorised as ‘Resources’, ‘Roles and Priorities’ or ‘Communication’. Specific factors identified were (1) Time and Funding, (2) Training and Materials, (3) Professional Roles and Priorities, (4) Personal Roles and Priorities, (5) Message Consistency and Clarity and (6) Supportive Relationships and Communication Styles. Within each factor, subcategories describing specific anticipated barriers and proposed enablers were identified. These mapped to the TFA constructs of Affective Attitude (four barriers, four enablers coded), Perceived Effectiveness (four barriers, three enablers), Self-Efficacy (three barriers, four enablers), Burden (three barriers, one enabler), Ethicality (one barrier, one enabler) and Opportunity Cost (one enabler). No data were coded relating to Intervention Coherence, or the extent to which participants understand the intervention and how it works. HCP had mixed opinions regarding the acceptability of delivering infant feeding interventions within vaccination visits. GP were predominantly in favour of this, with practice nurses somewhat evenly split between those who felt it would work *v*. those who felt it would not. PHN, community dietitians and community medical officers felt that it would be acceptable if the identified barriers were addressed.

**Table 2:** Proposed factors and barriers/enablers influencing the delivery of interventions to promote healthy infant feeding within primary care

Factors influencing the acceptability of delivering infant feeding interventions in primary care		Anticipated barriers to delivering infant feeding interventions in primary care (TFA construct coded)	Proposed enablers to delivering infant feeding interventions in primary care (TFA construct coded)
Resources:	Time and funding	• Lack of staffing capacity (i.e. time/funding) to deliver intervention (B) • *Increased recent complexity and therefore time needed for vaccination visits* * (B, PE)	• Incentivising involvement via audit, payment/staffing, free training, etc (B, OC) • *Vaccination visits providing ease of access to child at routine periods* * (PE)
	Training and materials	• Lack of available training/trustworthy resources (SE)	• A recognised need for more infant feeding support/training/resources for parents (SE) • Access to training/trustworthy resources for HCP (SE)
Roles and priorities:	Professional roles and priorities	• Too many competing priorities within existing HCP role (B, AA) • Childhood obesity/infant feeding not perceived as part of HCP role (AA)	• Multidisciplinary team involvement – viewed as an issue where all have a role to play (AA)
	Personal piorities	• Childhood obesity/infant feeding not in HCP’s personal area of special interest (E, AA)	• Perceived importance of childhood obesity/infant feeding – long-term social/economic implications (E, AA) • HCP having existing special interest in/personal experience with childhood obesity/infant feeding (E, AA)
Communication:	Message consistency and clarity	• Lack of message/information consistency across HCP (PE) • Conflicting information from other influences (peers, media, etc) (PE)	• Ensuring consistency of messages/information across all HCP (PE)
	Supportive relationships/communication	• Challenges of communicating childhood obesity/infant feeding in a sensitive manner (SE, AA) • *Communication challenges due to increased parental stress/anxiety during vaccinations* * (SE,PE)	• Positive, supportive relationships/communication between primary care HCP and parents (SE, AA) • *Ease of communication due to decreased parental anxiety due to child not being medically ill at time of vaccinations* * (SE, PE)

TFA, Theoretical Framework of Acceptability; B, Burden; OC, Opportunity Costs; PE, Perceived Effectiveness; SE, Self-Efficacy; HCP, healthcare professional; AA, Affective Attitude; E, Ethicality.

* Italics denote barriers/enablers identified specific to CHErIsH vaccination visits.

In the next section, the term ‘HCP’ is used when similar views and attitudes were expressed by all types of HCP. Where views differed across provider type, this is explicitly stated.

### Resources

#### Time and funding

All HCP felt that time and funding constraints represented a substantial barrier to delivering interventions to promote healthy infant feeding, with many noting that current services across several primary care settings were overwhelmed and understaffed. This mapped to the TFA construct of Burden, that is, a perceived increased effort required to deliver interventions due to these constraints.

‘We’re here totally stretched to capacity, we would love extra funding to meet the service needs of people who are being referred to us.’ (HCP19, Dietitian)

Many spoke about contextual factors that had increased this pressure and lack of capacity. These included the introduction of a recent contract for free primary care visits for children under 6 years of age^(39)^, and a recent increase in the amount of vaccines required which added an additional level of complexity and time needed. As such, HCP felt that any intervention delivered during vaccinations would have to be very brief and/or incentivised in some way and minimise the TFA construct ‘Opportunity Cost’ by linking with service audit, payment for staff time, etc. Despite this, many HCP felt vaccination visits would be a good time to intervene, as they provide routine access to parents at several regular time points. This mapped to Perceived Effectiveness, that is, the specific aspects of the proposed intervention likely to ensure its effectiveness.

‘If it was connected to the nurses’ vaccination schedule, because parents already attend anyway at that point. That would be useful, I think. It would be the nurse during the vaccination schedule, that that topic can be brought up, and then information can be given.’ (HCP12, GP)

#### Training and materials

Mapping to the TFA construct of Self-Efficacy, all HCP identified the availability of suitable, trustworthy training and resources regarding infant feeding as crucial for ensuring that they had sufficient knowledge and skills to provide infant feeding interventions. For example, PHN and dietitians highlighted the importance of the infant nutrition training and reference pack delivered by dietitians to PHN as part of a national programme^(40)^. However, most HCP felt that there was a limited focus on nutrition and infant feeding within existing GP and practice nurse training. Several participants had access to booklets which they found valuable for improving their own knowledge and helping them to address healthy infant feeding with parents. However, some HCP raised concerns about industry-funded resources, and that the trustworthiness of resources would be an important factor influencing their participation in infant feeding research or intervention delivery:

‘If I get information, and there’s any [FORMULA FEEDING INDUSTRY NAME] written over it…I’d be much more likely to put it in the bin, than if it says [UNIVERSITY NAME]. If it comes from [HOSPITAL NAME], [UNIVERSITY NAME] or [UNIVERSITY NAME2], and there’s no industry connection, I’d be much more likely to trust it, to be comfortable relaying this information. Industry information, there’s too much bias.’ (HCP12, GP)

All HCP felt there was a widely recognised need for parental support in relation to breast-feeding and introducing solid foods. From a broader level, they felt this recognition would facilitate their ability to engage with and deliver infant feeding health promotion interventions in primary care, for example, via better buy-in from practice management. Specifically, the importance of social support and practical skills-based education/training for parents from a trustworthy source was identified. Group-based community classes or ‘weaning workshops’ were suggested by many as a good way to provide both social support and training on practical skills.

### Roles and priorities

#### Professional roles and priorities

A key barrier discussed by all was the varied roles and competing priorities of primary care HCP, which mapped to the TFA construct of Affective Attitude in relation to how HCP feel about being tasked to deliver the intervention. This barrier was closely linked to time and funding-related barriers and also mapped to Burden, as HCP felt that having so many areas of clinical care to cover within restricted time frames limited their ability to devote time to health promotion or infant feeding. Although several HCP (including PHN) discussed infant feeding as particularly relevant within the PHN role, it was felt that the competing priorities of elderly care and other aspects of the PHN role would make it difficult to sufficiently address infant feeding or deliver additional interventions.

‘There’s so much in public health nursing, you’re a jack of all trades really. You’re going in to an older man there, a 90 year-old man and doing a dressing…The problem is, and it’s the same for a dietitian, your workload is for everyone.’ (HCP1, PHN)

Community dietitians similarly felt that infant feeding was integral within their role, but that due to competing priorities they did not have sufficient capacity to deliver preventive-focused interventions and mostly had to prioritise complex clinical issues. Similar sentiments were echoed by GP in terms of competing priorities; however, GP viewed infant feeding as less within their remit.

‘The difficulty is the priorities. It’s the prioritisation of what is seen as a priority… It’s not that the children aren’t a priority. It’s just sometimes when you’re in a busy practice like say currently we have ridiculous scenarios where we have all sorts of 80-year-olds who we have to send to hospital and sitting in trolleys for three days, but this is the reality of it.’ (HCP18, GP)

Overall, multidisciplinary team involvement was highlighted as important for facilitating the delivery of infant feeding interventions in primary care. Participants felt that all primary care HCP had a role to play in childhood obesity prevention and the delivery of primary care-based infant feeding interventions, to ensure the consistency of messages and information across HCP. However, the extent of involvement and perceived roles varied. Many HCP (including PHN and dietitians) felt that given their interaction with infants and parents at relevant times, PHN had a lead role – with the essential support of, and in collaboration with community dietitians. In relation to delivering a brief intervention at vaccination visits, they felt that practice nurses were appropriately placed to lead the delivery of this specific intervention.

‘It would be multidisciplinary; I would look at it, because that would give a bigger loop of support and education. So you would have I suppose practice nurse, GP, PHN. If concerns are going to develop, they would have a link to paediatrician and other services, if that was necessary….If it was to be in a primary care setting, you would have to have everybody involved in primary care on board.’ (HCP13, Practice Nurse)

#### Personal priorities

Having an existing interest in breast-feeding or infant feeding or personal experience of having children was mentioned by almost all HCP as a factor facilitating the delivery of infant feeding interventions within primary care.

‘Some people just have more interest in child health. Like in public health, because our range is so broad, some people have mentors in child health, some it’s elderly, some people it’s wound care or whatever. So if it’s in the PHN’s own interests.’ (HCP2, PHN)

All HCP felt that infant feeding was an important issue and were enthusiastic about the need to improve healthy feeding behaviours and prevent childhood obesity. This mapped to the TFA construct of Ethicality in relation to the intervention ‘fit’ with their personal value system, as well as Affective Attitude.

‘I think we are looking at a growing problem with obesity in children in Ireland and I think if it’s dealt with at grass roots level, at primary care, if infant feeding is discussed primarily in the early stages, in the long-term you are addressing obesity if you are looking at infant nutrition. So I think it’s of vital importance.’ (HCP1, PHN)

Several participants felt that explicitly highlighting this importance of infant feeding and the long-term economic and social implications of childhood obesity would be important to facilitate engagement of primary care HCP in delivering interventions to promote healthy infant feeding.

‘When your under sixes have healthier practices, through nutrition and through optimal infant feeding practices, then their health is much better, so it means that the burden to society and the practice is less…Then the practices are much more likely to go with it because it makes sense. It’s feasible. The extra work involved would be well worth it because it would actually give them more time to be able to deal with other patients sometimes that are more needy.’ (HCP5, GP)

### Communication

#### Message consistency and clarity

Several HCP highlighted the importance of consistency and clarity of infant feeding information across HCP, though many felt that this currently was not always the case.

‘I think everybody should be singing off the same hymn sheet when it comes to infant feeding but I think in practice that isn’t the reality.’ (HCP11, Practice Nurse)

HCP also felt that ensuring consistency across HCP delivering any infant feeding intervention was particularly important to counteract the mixed messages that parents encounter externally from other potentially inaccurate sources on parents’ feeding behaviours, for example, the media, books, peers and family members. This mapped to Perceived Effectiveness in terms of the perceived detrimental effect of mixed messages on intervention impact.

‘…there’s so much information out there in the media and the internet and everything, everyone thinks that they’re an expert in nutrition and it’s to try and make sure that people are getting the right messages.’ (HCP19, Dietitian)

#### Supportive relationships and communication

The communication and relationship between HCP and parents were identified by almost all HCP as a critical factor influencing the delivery of an infant feeding intervention in primary care. Some HCP felt that infant feeding was a potentially sensitive topic and sometimes challenging to raise with parents. As such, good communication skills and a supportive relationship between the HCP and parent were viewed as an important enabler, mapping to Self-Efficacy. In relation to delivering an intervention within vaccination visits, many HCP felt that existing relationships and trust between parents and practice nurses would actively facilitate this, and that parents could be more ‘receptive’.

‘The practice nurses, they have been probably one of the best resources in the general practice. I would say that that would be potentially a very positive piece because they have the benefit of ongoing relationships with the families and usually they’re very positive relationships and there would be a real sense of trust.’ (HCP14, Community Medical Officer)

However, HCP views differed regarding how well practice nurses would be able to communicate with parents during vaccination visits. Mapping to Perceived Effectiveness, many HCP, including practice nurses themselves, felt that practice nurses’ ability to communicate with parents could be hindered by increased parental stress/anxiety levels regarding the vaccinations and render parents less receptive to any additional information regarding infant feeding.

‘The problem is, what will they remember? If they are highly stressed coming into you, which most of them, definitely first vaccinations, all the mothers, parents, grannies, grandfathers, they all hate it. So, trying to give them any meaningful information at that visit, you’re trying to get across, vaccinations are a good thing. [laughing] You’re trying to tell them about all these diseases, and if you throw in nutrition on top of that, it would be overwhelming.’ (HCP10, Practice Nurse)

On the other hand, other HCP (including practice nurses) discussed the fact that infants attending for vaccination visits are medically well; therefore, parents would be less anxious/stressed and more receptive to infant feeding information.

## Discussion

This study is one of few qualitative studies to explore HCP’s views of the prospective acceptability of delivering early-life childhood obesity prevention interventions within primary care settings. To the best of our knowledge, it is also the first to specifically explore HCP’s views of an opportunistic childhood obesity prevention intervention targeting infant feeding within routine vaccination visits. Drawing on existing experience, HCP identified a number of factors that would influence how acceptable they would find the delivery of interventions to promote healthy infant feeding within primary care settings. These were the availability of trustworthy resources for parents and HCP, HCP’s roles and associated priorities, and factors relating to communication and relationships between HCP and parents. Proposed barriers and enablers to delivering interventions within vaccination visits include time constraints *v*. opportunistic access to ‘well’ children, existing relationships and trust between parents and practice nurses and potential communication issues during the vaccination visit. When mapped to the TFA, barriers and enablers relating to how HCP feel about the intervention, its Perceived Effectiveness and their own Self-Efficacy were the most frequently coded. This may highlight priorities and broad constructs of acceptability to consider targeting in developing infant feeding interventions for delivery by primary care HCP.

This study also emphasises the need for clear role delineation and ownership regarding the prevention of childhood obesity in primary care. Childhood obesity prevention was viewed by participants as an important societal issue where all primary care providers have a role to play, and that coordination of roles across HCP is important to ensure consistency. However, what exactly these roles should entail and the respective ownership of roles between HCP was less clear. This corresponds with the findings of Redsell et al. who found that GP and practice nurses predominantly viewed childhood obesity prevention and provision of infant feeding advice as beyond their remit^(29)^. Similarly, Pearce et al. (2018) found that many HCP do not view obesity prevention as a core part of their role and identified a mismatch between current prevention-focused health policies and the existing reactive medical model which has shaped clinical practice^(41)^. Recent times have seen an increasing global focus on chronic disease prevention and a shift in emphasis from reactive healthcare systems towards preventive care^(42)^. As such, HCP roles and ownership for different facets of healthcare may need to change and evolve accordingly. However, if this is to be achieved, the findings of this study suggest that clarity regarding the content, ownership and leadership for new roles is crucial, and that redefinition of roles needs to be done in collaboration with HCP and coordinated across different individuals and actors within the system. This would also need to carefully consider adequate resource allocation, as time and staffing constraints were a significant concern for participants in terms of their ability to prioritise public health promotion. As such, resources available to HCP would also need to evolve and change to reflect any new roles, responsibilities and altered priorities.

The need for adequate resources in the form of time, funding, training and supporting materials was one of the most commonly discussed factors influencing the delivery of an infant feeding intervention within primary care. This is unsurprising, as a lack of resources are often identified as a barrier to intervention delivery by HCP within routine care settings^(28,43)^. However, the explicit requirement for trustworthy and unbiased training and educational resources in this study is of particular note. Our findings show that primary care HCP require adequate training and supporting materials if they are expected to deliver infant feeding advice to parents. However, in line with previous research^(29,44)^, we identified deficits in current HCP pre-registration training in relation to infant feeding. If sufficient training is not available from impartial sources, then it is possible that many HCP may resort to attending industry-sponsored training to fill these resource gaps, as previously found in a systematic review of the interactions between HCP and industry^(45)^. Issues pertaining to conflicts of interest and the influence of nutrition industry funding within health research and clinical practice have come under increased scrutiny in recent times^(45–50)^. The 2016 WHO Guidance on Ending Inappropriate Promotion of Foods for Infants and Young Children specifically prohibits the sponsorship of meetings and the ‘provision of information for health workers other than that which is scientific and factual’^(51)^. However, a 2018 WHO report showed that international implementation of this guidance remains subpar with much room for improvement^(52)^. Therefore, there is a duty to provide access to unbiased training and education for HCP to facilitate delivery of infant feeding interventions within primary care settings, for transparency regarding funding sources, and not to expect or enable existing health service resource gaps to be filled by industry.

Our findings also allude to the importance of taking a collaborative, systems-level approach towards childhood obesity prevention, particularly when it comes to utilising vaccination visits as an opportunistic time point for brief behaviour change interventions. Similar to previous explorations of primary care HCP’s views on childhood obesity management^(28)^ and brief opportunistic behaviour change interventions^(24)^, HCP in our study identified the need to consider not just individual HCP-level factors influencing delivery but also broader factors. These included allocation of health service resources and the consistency of public health messages across multiple levels of actors including HCP beyond primary care, professionals beyond healthcare (e.g. education) as well as social media and the wider public. In addition, participants expressed mixed opinions towards the idea of using vaccination visits for a brief behaviour change intervention, and many discussed the potential of group-based ‘weaning workshops’ delivered in primary care. Previous research has highlighted the potential of brief behaviour change interventions delivered by primary care HCP^(24,53–55)^; however, much of this research has targeted adult behaviours such as physical activity, smoking, diet and alcohol use. Aiming to improve infant nutrition and childhood obesity by targeting parental feeding behaviours adds an extra level of complexity and therefore may require a broader and more concerted effort across multiple HCP and systems, for example, supplementing brief interventions with ‘weaning workshops’, addressing infant feeding marketing/advertising and availability of healthy foods. This echoes the findings of a recent scoping review on the role of hospital and community-based services in obesity prevention which also highlighted the need for a systems-level approach to explore the perspectives of a range of players within the system and consider the multifactorial causes of obesity and subsequent opportunities for intervention^(41)^.

### Strengths and limitations

This study included a varied sample of primary HCP from GP to community dieticians, with efforts made to recruit participants across a spread of ages and genders, with and without children. However, other categories of HCP such as community midwives could have been included, and there were unequal numbers recruited in different HCP categories which should be considered in interpreting and generalising study findings. This may have potentially biased findings by giving undue prominence to views of certain HCP groups, as different HCP may have different experiences and perceptions based on their perceived or actual role in engaging with parents around infant feeding. However, efforts were made during the analysis and presentation of findings to ensure that where views differed across provider type, this was explicitly considered and stated. Additionally, given the importance of a systems-level perspective and organisational-level factors identified within the study, other personnel such as healthcare managers and primary care clinic administrators may also have provided additional important insights. Additionally, this study focused on prospective acceptability of interventions to promote healthy infant feeding in primary care. HCP naturally drew on existing experiences to inform their views of potential barriers and enablers; this sometimes made it difficult to fully understand the current landscape and disentangle actual barriers and enablers from hypothetical issues or recommendations.

This study is one of the first studies to use the TFA^(32)^ to prospectively explore specific constructs of acceptability for intervention development. Applying the TFA facilitated a structured and comprehensive approach to exploring intervention acceptability prior to implementation. This has enabled us to anticipate and address potential issues in advance to support intervention delivery and will facilitate retrospective comparisons of experienced acceptability issues afterwards, as well as comparisons with other research studies that similarly use the TFA. However, the TFA is still in its infancy, and there is a need for more clarity and examples of framework application. For example, we experienced some ambiguity between constructs of Affective Attitude and Perceived Effectiveness, similar to challenges identified recently by Pavlova et al. in applying the TFA^(56)^.

## Conclusion

This study provides a unique insight into the delivery of infant feeding interventions to prevent childhood obesity from the perspectives of a variety of primary care HCP. With an increasing global focus on preventive care, such interventions have substantial promise; however, a number of factors across both HCP and systems levels should be considered in advance to ensure maximum acceptability of interventions to providers. These include a collaborative and coordinated approach to intervention delivery across all HCP and all relevant systems-level actors including policymakers to ensure sufficient resourcing for HCP, both in terms of unbiased training and educational resources, as well as adequate staffing and funding, and clarity regarding roles and ownership within a preventive care lens.

Overall, our study highlights that policy development needs to be cognisant of the current systems, structures and resources, and in particular, of how these will fit with existing roles and priorities for HCP. As such, childhood obesity prevention policy and associated interventions need to be developed in collaboration with stakeholders, not only with the parents and families of young infants, but also with the HCP who will be involved in rolling out and implementing those policies and interventions. Future research also needs to consider the involvement of stakeholders across multiple levels and to develop and evaluate more sustainable interventions by focusing on implementation from the outset, for example, using hybrid trial designs that concurrently test the effectiveness of interventions and associated implementation strategies^(57)^. Finally, primary care practice provides a unique opportunity to influence long-term health and societal outcomes, and as one GP stated, *‘You can make a lifelong change. This is probably the least input for the biggest output, if you do something then* [in infancy], *it’s fantastic’*. For HCP, a challenge remains to stay open to changing roles and priorities and to work with researchers and policymakers to identify realistic ways of overcoming the barriers identified in our study to achieve maximum impact from early-life intervention.

## Supplementary material

For supplementary material accompanying this paper visit https://doi.org/10.1017/S1368980020004954. click here to view supplementary material
