# Supplementary material for: Exploring healthcare professionals’ views of the acceptability of delivering interventions to promote healthy infant feeding practices within primary care: a qualitative interview study
Source: Public Health Nutr. 2020 Dec 15;24(10):2889–99. doi: 10.1017/S1368980020004954 (PMC9884767; doi:10.1017/S1368980020004954)
Supplement: Supplementary file 1 [file S1368980020004954sup.zip › urn_cambridge.org_id_binary_20210107050838722-0929_S1368980020004954sup001.docx]

**Theoretical Framework of Acceptability Coding**

**Coding instructions:**

1) Code each barrier and enabler for TFA category (if applicable) using brackets and abbreviations for TFA category (e.g. Affective Attitude = AA)

2) For factor, put overall categories coded, e.g. AA and B if both Affective attitude and Burden are coded for enablers/barriers within that factor.

3) Don’t code if TFA category not relevant

**Consensus process:**

1) Compare coding – highlight any discrepancy

2) Discuss categories and perceived meaning within this intervention

3) Discuss specific discrepancies revisit coding mutually within discussion

4) Consensus achieved for final codes


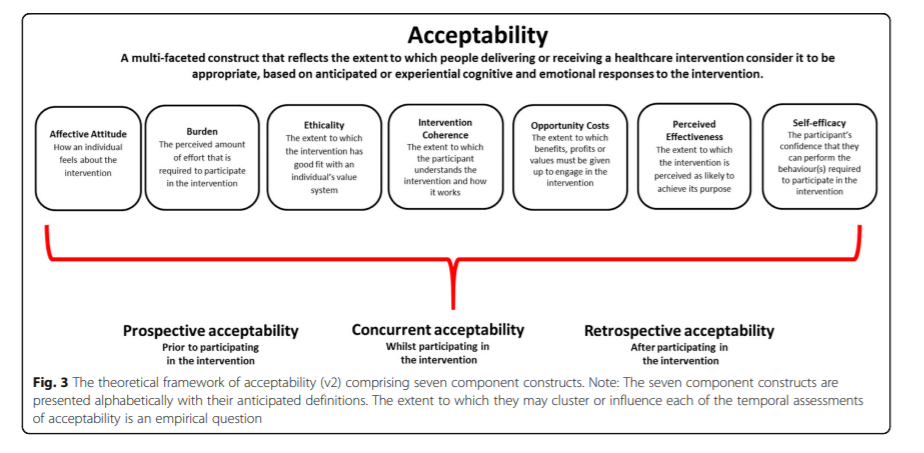


Sekhon et al 2017

**Codebook:**

| **Affective Attitude** –HCPs perceptions of the importance of the intervention, how they feel about being the ones tasked to deliver the intervention, e.g. it being their role or not  **Burden** – HCPs perceptions about effort to participate in intervention due to staffing, funding limitations, their existing roles and priorities etc  **Ethicality** – HCPs’ personal views about the importance of this topic, and how this intervention is important and fits with their personal value system  **Opportunity Cost** – HCPs thoughts on how to balance time/resources required with potential for gain (i.e. minimising opportunity cost)  **Perceived Effectiveness** – Specific aspects of this intervention that HCPs perceived would probably impact on intervention effectiveness  **Self-Efficacy** – HCPs’ confidence about their own level of knowledge and skills eg communication needed to deliver the intervention |
| --- |
