# Supplementary material for: Exploring healthcare professionals’ views of the acceptability of delivering interventions to promote healthy infant feeding practices within primary care: a qualitative interview study
Source: Public Health Nutr. 2020 Dec 15;24(10):2889–99. doi: 10.1017/S1368980020004954 (PMC9884767; doi:10.1017/S1368980020004954)
Supplement: Supplementary file 1 [file S1368980020004954sup.zip › urn_cambridge.org_id_binary_20210107050846537-0285_S1368980020004954sup002.docx]

**Audit trail of analysis steps**

1) Read and reread transcripts for familiarisation

2) Initial brief overview coding data relevant to interview aims and research question – deductive approach:

a) Key points under the following headings (taken from aims of interview and key questions from topic guide) summarised for each HCP and tabulated:

- - Experience of infant feeding
  - Specific training
  - Role of HCP in infant feeding
  - Ideal IF intervention
  - Vaccination visit feasibility (opinions for, against etc)
  - Barriers to HCP involvement
  - Facilitators to HCP involvement
  - Other

b) Each section above summarised to identify key findings from all interviews for above sections, which led to the generation of the following **initial codes**:

- - *Importance of topic*
  - *Need for parent training/social support/trustworthy resources*
  - *HCP roles and MDT involvement*
  - *Access to parents*
  - *Need for trustworthy HCP resources/time/capacity*
  - *HCP/parent relationships/communication*
  - *Consistency between/across HCPs*
  - *HCP personal interest/knowledge/experience*
  - *External parental factors eg stress/emotions*

3) Initial line by line coding of all raw data from transcripts done in NVIVO into the codes identified above, organising them according to:

- General acceptability of addressing infant feeding within a primary care context
- Specific barriers and enablers to delivering infant feeding interventions during vaccination visits (within each code)
- Additional codes if needed

4) Two transcripts sent to CF for coding into the codes above, discussion of coding by ET and CF

4) For each initial code above, reviewed data in each code and codes themselves and collated into **initial themes** for each – e.g. overarching concepts, e.g. split up *Need for trustworthy HCP resources/time/capacity* into *Training/Resources* and *Time/Funding,* and merged *‘Need for parent training/social support/trustworthy resources’* with *‘Need for trustworthy HCP resources/time/capacity’ into Training/Resources*

- *Topic importance*
- *Training and Resources*
- *Time and Funding*
- *Roles and Priorities*
- *Communication and relationships*
- *Message consistency*

5) Summarised the findings for each initial theme across all HCPs and wrote up narratively (Results Overview)

6) Sent Results Overview (detailed overview of initial themes and findings) to CF for review and discussion

7) After CF review and discussion revised and restructured initial themes into **final themes** (representing the broad factors that influence delivery of infant feeding interventions) and subthemes (specific enablers and barriers):

- **Resources:** Time and Funding, Training and Materials
- **Roles and Priorities:** Professional Roles and Priorities, Personal Roles and Priorities
- **Communication:** Message Consistency and Clarity, Supportive Relationships/ Communication Styles

8) Generated final table of findings (in Results Document) based on final themes according to

- Factors influencing the implementation of infant feeding interventions in primary care
- Barriers to implementing infant feeding interventions in primary care
- Enablers to implementing infant feeding interventions in primary care, and marked specific barriers/enablers to CHERISH/vaccination visit idea.

9) Table of barriers/enablers coded according to TFA by ET – sent to EO for second-rater coding
