# Supplementary material for: Exploring healthcare professionals’ views of the acceptability of delivering interventions to promote healthy infant feeding practices within primary care: a qualitative interview study
Source: Public Health Nutr. 2020 Dec 15;24(10):2889–99. doi: 10.1017/S1368980020004954 (PMC9884767; doi:10.1017/S1368980020004954)
Supplement: Supplementary file 1 [file S1368980020004954sup.zip › urn_cambridge.org_id_binary_20210107050854462-0187_S1368980020004954sup003.docx]

| Initial interview/focus group warm-up:   - Introductions - Reiteration of purpose of interviews/focus groups e.g. looking to develop an approach that can be used in primary care in the early infancy period to help shape and improve how parents feed their children during that period (0-2). |
| --- |
| **Aim 1:** Identify barriers and enablers to participating in a brief intervention to promote healthy feeding practices for HCPs  **Aim 2:** Explore experiences of and attitudes towards intervention components to determine their feasibility and acceptability for HCPs |
| Opening questions:   - Can you tell me about your own background in terms of infant feeding? (e.g. experiences, understanding, specific training, attitudes, opinions on infant feeding) PERSONAL/PROFESSIONAL/BOTH? - What do you think is the role of healthcare providers in infant feeding? Or the role of healthcare providers in infant feeding interventions? - What are your attitudes towards being involved in an infant feeding intervention? Which HCPs (if any) do you think should be involved? - What do you think an intervention to promote healthy infant feeding in parents of young children in a primary care setting should involve?   - Vaccination visits? Feasibility? - In your opinion, what do you think are the main things that would influence healthcare providers’ participation in an intervention that aimed to promote healthy infant feeding behaviours? |

**Appendix 1: Topic guide for interviews/focus groups with healthcare providers (HCPs)**
